# Inter-basin surface water transfers database for public water supplies in conterminous United States, 1986–2015

**DOI:** 10.1038/s41597-023-02148-5

**Published:** 2023-05-06

**Authors:** G. Rebecca Dobbs, Ning Liu, Peter V. Caldwell, Chelcy Ford Miniat, Ge Sun, Kai Duan, Paul V. Bolstad

**Affiliations:** 1 Southern Research Station, USDA Forest Service, Asheville, USA; 2 Oak Ridge Institute for Science and Education, Oak Ridge, USA; 3 Commonwealth Scientific and Industrial Research Organisation - Environment, Canberra, Australia; 4 Rocky Mountain Research Station, USDA Forest Service, Albuquerque, USA; 5 Sun Yat-sen University, Guangzhou, P. R. China; 6 University of Minnesota, Minneapolis, USA

**Keywords:** Hydrology, Water resources

## Abstract

The manipulation of water resources is a common human solution to water-related problems. Of particular interest because of impacts on both source and destination is the anthropogenic movement of water from one basin to another, or inter-basin transfers (IBTs). In the United States, IBTs occur widely in both wet and dry regions, but IBT data are not collated and served in a coordinated way. Thus researchers wishing to account for transfers between basins have faced difficulty in doing so. Here we present the outcome of a systematic investigation into inter-basin surface water transfers connected with public water supplies in the conterminous United States (CONUS), 1986 to 2015. The present open-access geodatabase includes transfer volumes collected, evaluated, and compiled from disparate sources. We provide an updated snapshot of CONUS IBTs at a higher spatial resolution of points of withdrawal and delivery than previous datasets. This paper puts the national inter-basin transfer data in context, and shows how we acquired, structured, and validated the locations and volumes of surface water transfers in public water systems.

## Background & Summary

Human societies broadly, through time and on every inhabited continent, have moved or otherwise managed water in order to support urban populations, moisten dry places, modify watery landscapes, make water available during dry times, ease the transport of people or goods, or improve access to aquatic resource species^1–19^. As Ersten^20^ notes (p. 165), transferring water “requires both physical distribution facilities to transport water and socio-political arrangements to coordinate between actors in dealing with water flows”, and so tends to be associated with a level of governance capable of coordinating both labor and water allocation (i.e., a State; but compare ref. ^21^). With the rise of the industrial revolution, new processes, tools, and materials made possible an age of “modern” inter-basin transfers (IBTs) beginning in the 19^th^ century^22^, one which required some additional levels of coordination from governments across scales from local to international. The role of the United States (US) federal government in planning, building, and operating large IBT projects in the western US was particularly important during the 20^th^ century. Despite this, there is no ongoing centralized collation or archiving of water transfer data by the US government, in part because multiple disparate agencies were involved at multiple levels of government, accentuating both the need for and the difficulty of compiling a comprehensive public water supply IBT database such as we present here.

**Fig. 1:**
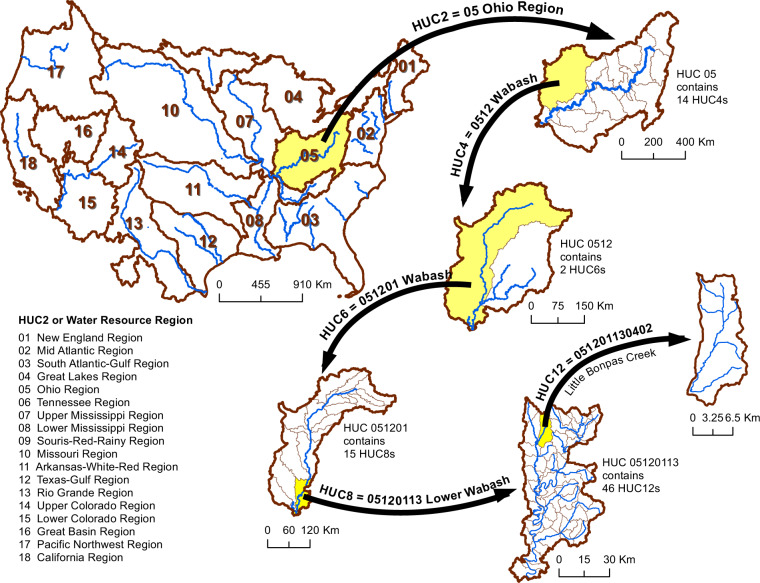
Representation of USGS Hydrologic Unit Code system (in CONUS).

IBT studies focused within the US^23–26^ have largely relied on a two-volume dataset created by the US Geological Survey (USGS) in the 1980s^27,28^. For this dataset state-level USGS officials were asked to fill out survey questionnaires about IBTs that were active and ongoing in 1982, and to include estimated annual volumes transferred during 1973–1982. IBTs were defined as transfers crossing “subregion” watershed boundaries at the 4-digit hydrologic unit code (HUC4) scale (see Fig. 1 and Methods section) to limit the number of transfers in the inventory, while donor and recipient watersheds were identified at the 8-digit (HUC8) scale. For the western dataset^28^, 16 state USGS offices were surveyed, while for the eastern dataset^27^, 18 were surveyed. Thus one weakness of the resulting combined dataset is that a large area in the central CONUS was not surveyed. Further, only those received responses containing the “most complete” (ref. ^27^, p. 3; ref. ^28^, p. 4) data were compiled in the two published reports. Nevertheless, for researchers wishing to adapt their hydrologic modelling to include IBTs this has long been the best available data source. A more recent inventory^29^ is based on the presence of infrastructure rather than the functional use and includes a much higher number of potential transfers but does not supply any flow volumes for incorporation into modelling.

The volume of IBT research overall increased steadily from the early 1990s to 2014, and most especially so in China, the US, Australia, and to a lesser extent Canada, toward the end of that period^30^. Many early works focused on ecological changes resulting from IBTs^22,31–35^, but researchers have also examined social effects of IBTs through lenses such as political ecology^36^, political economy^37^, and cultural politics^38^, as well as the political and economic drivers of IBTs^39^. As issues of climate change and the sustainability, or otherwise, of human water engineering become ever more pressing, IBT research at every scale is increasing in importance and increasingly focused on the nexus of human population growth, environmental needs for water, and the changing availability of water under accelerating climate change^25,26,40–46^. Our work adds to the existing body of literature on IBTs in the US by providing an update to the important but now-dated inventories created by the USGS^27,28^, improving spatial resolution of points of withdrawal and delivery, and adding detail to spatially complex IBT systems, and will support future research of a critical nature under current difficult conditions.

Here we present a new database of inter-basin surface water transfers, including flow volumes, for public water supplies in the conterminous United States (CONUS) from 1986 through 2015^47^. Our immediate goal in building this database was to provide IBT data for incorporation into modelling of surface water originating in National Forests and other forested lands and delivered to Public Water Systems (PWSs)^43^. IBT research by others will also benefit from the much-needed update of data and higher spatial resolution of origin and destination HUCs in this new database. We anticipate that researchers will adapt and extend our database to help support such critically important IBT research in the CONUS and elsewhere around the world. Below we discuss methods of data acquisition, structure and characteristics of data records, and validation of the database.

## Methods

### Scope and purpose

The spatial scope of our work is the CONUS, or the “lower 48 states” of the US. As such our inventory of IBTs is more comprehensive than that performed by the USGS in the 1980s^27,28^ in that we investigated all 48 of the conterminous states (although the inventory in ref. ^29^ does cover all of CONUS). The spatial resolution of our IBT data is finer than that in those previously published inventories, as well, thus supporting more detailed hydrologic modelling. Further, our database represents visually and in spatial context the complex topologies of water collection and delivery in IBT systems.

The temporal scope of the work includes the years 1986 through 2015. This ideally yields a 30–year data collection, though in reality, transfers might have come online later than 1986 or ceased functioning before 2015, or agencies might not have retained earlier data. All data received were aggregated to the annual timestep and standardized for units (Mm^3^ water yr^−1^), then compiled into the database where they are presented in a uniform and documented format.

The database is extensible in that users can enter additional transfers, add more years, or change the time step. The database structure could also be adapted for non-US transfers by utilizing watershed frameworks available for use in the target country. We expect that researchers will find the database of use both as a set of data of defined scope, and as a structure useful when extending that scope.

### Defining IBT criteria

At its most fundamental, an IBT is an anthropogenic movement of water from its natural source basin to some other basin. The term “basin”, however, as an area of land where natural flow accumulates to the single outlet point where it exits the basin, can thus be understood at very different scales. In the US, a formal system for the identification of basins at different scales has been derived by the USGS. As illustrated in Fig. 1, each low-digit basin or hydrologic unit is comprised of numerous higher-digit basins or units nested within it. Thus to speak of IBTs in terms of water crossing HUC4 (4-digit) boundaries results in a coarser spatial resolution for the data than that crossing HUC8 (8-digit) boundaries.

**Fig. 2:**
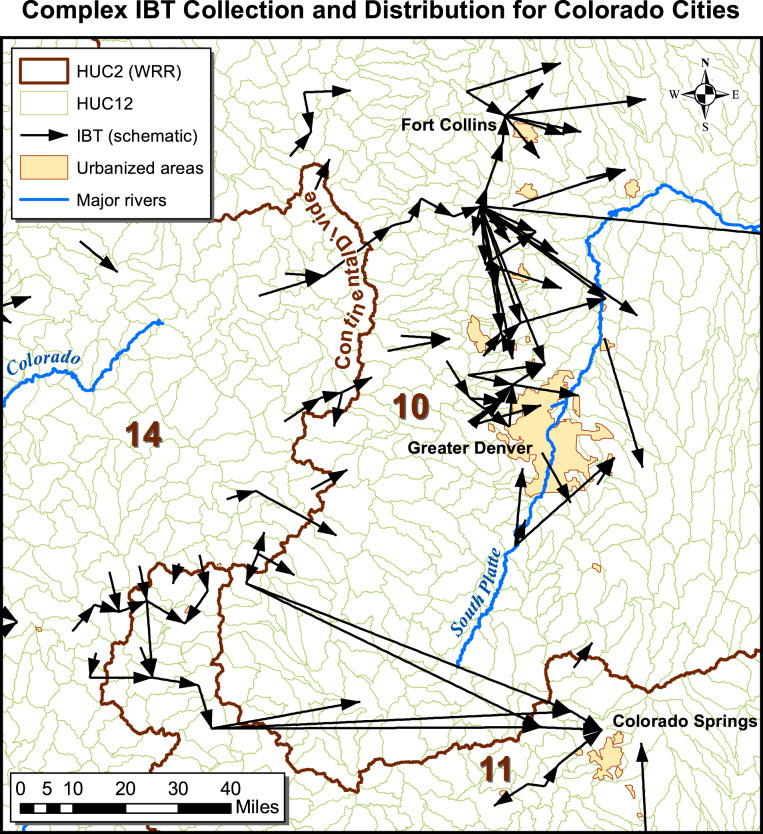
These complex water collection and distribution systems on the western and the eastern of the Continental Divide in Colorado illustrate the need for HUC12-scale IBT data in certain locations.

The first criterion to establish was therefore the scale of hydrologic unit at which to define and represent IBTs. As we wished to use the data in finer resolution hydrologic modelling than was possible with the USGS IBT data, we established a default scale of HUC8 for the boundary the water must cross to be considered an IBT, while recognizing that in some instances we would need to employ a finer scale in order to fully resolve the IBT connections. For example, in Colorado many significant water transfers begin by collecting snowmelt from mountain stream headwaters in multiple HUC12s west of the Continental Divide, interconnecting at various points, and ending in a complex array of HUC12s east of the Continental Divide (Fig. 2). California likewise has complex and interlocking systems best understood at the HUC12 scale. Furthermore, for every transfer in the database we identified origin and destination watersheds at the HUC12 scale, to support high-resolution modelling.

We next established criteria regarding water type, use type, and endpoints. We chose to include only transfers originating in surface water, because we could not fully account for the origin location of any groundwater source. Most of our IBTs end as surface water as well, although as cities and states attempt to restore overused groundwater sources, transfers of surface water increasingly end at groundwater infiltration facilities; in instances where we acquired data for in-ground deliveries, we included them in the database (notably in Arizona). We chose to define drinking water transfers as ending at water treatment plants (WTPs) rather than accounting for domestic uses and water returns such as wastewater treatment plant effluent. This decision has some ramifications in that a WTP may be in a different HUC8 from the population served, such that an IBT exists in effect but is not recorded in our database; conversely, if a source and consuming population are in the same HUC but the WTP is in another, an IBT exists in the database though not in effect. Given our focus on drinking water supply, we did not seek out transfer systems where the sole purpose was irrigation, but did include irrigation transfers where domestic and agricultural transfers were part of the same overall systems. Where irrigation transfers were included, we defined them as ending at the terminus of conveyances visible on maps or in the National Hydrography Dataset (NHD; https://www.usgs.gov/national-hydrography/access-national-hydrography-products) geospatial data, if no final delivery point was defined in the tabular data source. We did not include consumptive transfers for industrial uses such as thermoelectric plants; water transferred to a hydroelectric plant, however, was considered to continue onward as a transfer after passing through the plant’s equipment. We also did not fully account for water exchange agreements (common in the western states), but the database is designed such that they can be added by users or in a future version.

Our final criterion considered the population served by a Public Water System (PWS). If a city was not connected to a complex system of IBTs such as those mentioned in Colorado and California, we limited our defined IBTs to cities whose PWSs served populations of at least 200,000 based on the 2017 Safe Drinking Water Information System (SDWIS; https://www.epa.gov/ground-water-and-drinking-water/safe-drinking-water-information-system-sdwis-federal-reporting) data from the US Environmental Protection Agency (EPA), *and* the PWS received surface water from across a HUC8 boundary as discussed previously. Cities of this size are often important centers of function and culture for less urbanized states or regions, or they may form important parts of larger metropolitan conglomerations. In either context, they are likely to have a greater per capita water demand due to development trajectories than small cities and towns, and they often sell water to smaller nearby PWSs as well. Using this criterion, we included, for example, Tulsa, OK (population served about 471,000) and Cincinnati, OH (population served about 749,000), but not Wichita, KS (population served about 390,000) because the water sources were groundwater, or Rochester, NY (population served about 214,000) because the water sources are in the same HUC8 as the WTP.

### IBT data acquisition

The first step in IBT data acquisition was to find and develop an understanding of the transfers existing in the CONUS. We used three approaches to do this. First, we looked at major cities’ utility organizations to see which PWSs indicated they used water sources away from the city. Many large PWSs provide generalized system maps or descriptions for public use, and these helped us connect sources to urban WTPs. Second, we investigated known major water infrastructure projects (e.g., Central Arizona Project [CAP], California’s State Water Project [SWP]) that supply multiple urban and non-urban locations with water from IBTs. Such projects sometimes have their own websites, or are described and illustrated on an agency site such as that of the US Bureau of Reclamation (BoR). Third, we filtered the 1980s USGS IBT data by our defining criteria to make sure we hadn’t missed any qualifying legacy transfers. Note that though we did add some transfers to our database from this third approach, the overall map of our dataset appears quite different from the USGS dataset. This is a consequence of our definition choices, differences in scale, differences in volume data sources, and changes in both natural hydrologic flows and anthropogenic transfer activity over the decades since the USGS data were collected.

Some IBTs are very straightforward, i.e., they can be schematically represented by a single arrow. In such cases we had only to identify the starting and ending HUC12s, by examining a variety of map, imagery, and textual materials. Many other IBTs are complex, however, with changes occurring between overall origin and destination such as introduction of additional transfer water, local deliveries, intermingling with water from multiple sources in a reservoir, or being discharged into a natural stream or river to be withdrawn again downstream. Since each such event modifies the flow volume and/or composition of an IBT’s water, it must be treated as a sub-transfer with its own origin, destination, and volume data, while also forming part of the system of which it is a part. In our geodatabase, we designate these sub-transfers as “steps”. In the case of complex IBTs, it was necessary to identify each step and its topological relationships with the rest of the system in addition to origin and destination HUC12s for each step. Represented schematically (via straight arrows from HUC centroid to HUC centroid, for each step), the topology of a complex system requires from two to dozens of arrows and associated pairs of HUC12s, worked out through detailed analysis of available source materials. Topologies were especially important for understanding complex systems that were interconnected with other systems. Greater Los Angeles, for example, receives water from multiple major aqueducts carrying water from the east, the northeast, and the north, much of which is distributed to different agencies, some of which is combined in different reservoirs and treatment plants, and some of those agencies also redistribute both wholesale and retail water to additional agencies, making for quite a complicated topology.

Sources for flow data also had to be researched extensively. While a smaller municipal system with a single IBT might readily provide data after a simple email request, others required that we submit Freedom of Information Act documents or explain the project more fully by telephone. Some municipal systems referred us to state-level water agencies, and many of the more complex IBTs required us to find and use data from multiple sources. The New York City system was especially tricky because some while most of the needed data were recorded by the USGS, some years were only in archived reports while others were available by data server; a final few had to be acquired through the NYC Open Records system. For major IBT projects, multiple agencies might be involved; in such cases records are often structured in terms of accounts (which agency owns how much water in the reservoir or pipe, for instance) rather than in terms of the movement of physical water from point to point. Once we knew what data to ask for, however, several large entities in the west, such as the CAP and the BoR’s Lower Colorado office, provided custom physical volume data at our request. In California, the Department of Water Resources provided us with access to historical annual SWP reports that supplement account-based reporting with tables of physical deliveries and pump station volumes. The State of Colorado serves downloadable tables for many of the diversion points in the state, providing physical volume data in some instances where we could not otherwise acquire any. In some other areas, transfer reports included physical volumes but only as spot checks. A summary of our data source agencies and their scales of operation can be seen in Supplementary Table 1, while the geodatabase itself details the source information for each transfer step.

Data were received from the various source agencies in a wide variety of time steps. Timesteps of flow volume data ranged from occasional spot checks to daily flow measurements, which had sometimes been aggregated to monthly and annual data reported on either a calendar or water year basis depending on agency policy. Values were aggregated to annual as needed and units converted to million meters cubed per year.

## Data Records

### IBT database structure

**Fig. 3:**
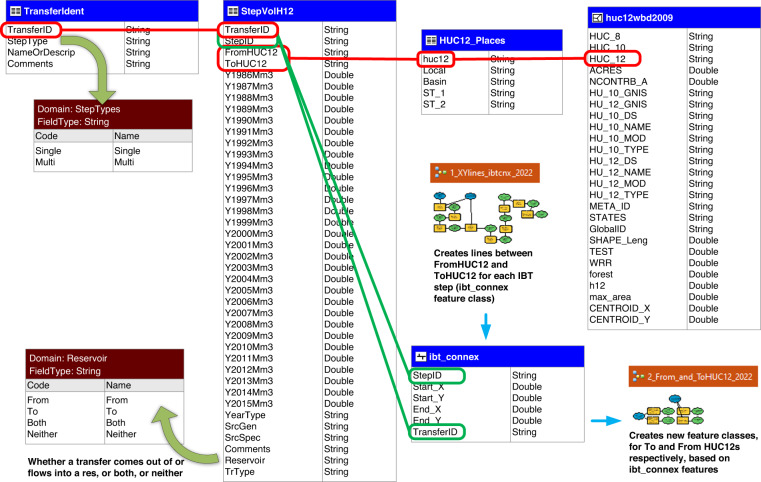
The structure of the IBT geodatabase. Tables, feature classes, domains, and models are shown, along with key relationships among them.

The IBT records reside in a geodatabase which has been deposited in the open data repository figshare^47^. The geodatabase (Fig. 3), contains tables, spatial feature classes, and a toolbox, which are discussed separately below. Functionality of the components is dependent on two interrelated systems of unique identification (ID) numbers. The first is the *TransferID*, consisting of five digits wherein the first two digits represent the Water Resource Region (WRR; also known as a 2-digit HUC) of the transfer’s origin. The remaining three digits of the *TransferID* reference a specific IBT or IBT system originating in that WRR, up to 999 such transfers. The *TransferID* data type is “text,” to avoid problems with leading zeros in the 01 through 09 WRR designations.

**Fig. 4:**
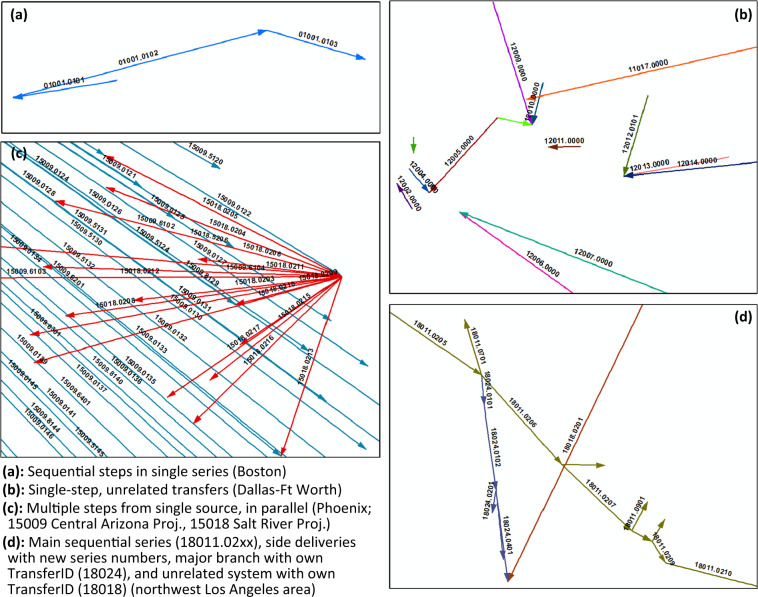
Step and *StepID* types. The steps of each *TransferID* share a color in the figure.

*StepID*, the second ID system in use, identifies internal steps comprising the transfer or transfer system designated by a given *TransferID*. As mentioned above, steps can be thought of as sub-transfers and so each step needs its own ID so that users can track and isolate water from a particular transfer at any point along the route between the transfer’s overall origin and destination. If the complete transfer consists of only an origin and a destination, with no intervening points where water is added, delivered, or altered in composition, it has only one step, and the *StepID* is “[*TransferID*]0.0000” (as in Fig. 4b). In contrast, a complex transfer may have multiple origins, multiple intervening points where water composition or volume is changed by anthropogenic or natural inputs or anthropogenic deliveries, and/or multiple endpoints. In these cases, the first two digits after the period represent a step series, while the final two digits represent a step within that series. If a transfer’s steps form a single linear arrangement (one origin, one or more intervening points, and one destination, as in Fig. 4a), the series designation is 0.01. In other configurations additional series in the same complex transfer are designated sequentially with 0.02, 0.03, and so on (as in Fig. 4d). Where there are multiple destinations but only one origin, as part of the same transfer system and without opportunities for water composition to change, such as in the Central Arizona Project, each origin-destination pair is designated as a step within one series (as in Fig. 4c). In contrast, where multiple deliveries originate in one source but are not integrated in a single transfer system, they will have different series designations or even different *TransferIDs*. Figure 4 and Table 1 illustrate these step numbering conventions, along with special cases where numbers other than zero are used to begin the series designation. As with *TransferID*, the *StepID* data type is “text” to avoid unintended decimal formatting or loss of leading zeros.

**Table 1:** ID conventions in the IBT data.

StepID			
TransferID			
Origin WRR	Assigned #	Step series	Step #
XX	XXX	.xx	xx
01 to 18	001 to 999	0.00 = IBT has only one step	00
		0.01 = first or only series, containing at least two steps in sequence*	01 to 99
		0.02 through 0.49 = additional series in same transfer system, each having at least one step	
		0.5x = an in-ground delivery for local groundwater recharge, with remainder of ID synced to normal step(s) in same transfer system	
		0.6x = same as 0.5x, but to a named underground water banking facility	
		0.7x not assigned	
		0.8x = lost water reported in data	
		0.9x not assigned	
		* Or, in parallel from same source, e.g. CAP canal in Phoenix	

The geodatabase contains three related tables. Of these, StepVolH12 contains the actual volume data, and is discussed in the next subsection. The two additional tables add value to the StepVolH12 table. The first, HUC12_Places, lists every HUC12 present in the step volume table and gives a description of that HUC12, usually in relation to features relevant to the transfers. The second, TransferIdent, lists every *TransferID* in the database and provides a description of the overall transfer. All three tables can be joined together, allowing for seamless descriptive and quantitative attribute data.

The geodatabase also includes certain spatial data for a user’s convenience. Although HUC12 (or other scale) polygons are readily available from the Watershed Boundary Dataset (WBD; https://www.usgs.gov/national-hydrography/access-national-hydrography-products), those polygon borders and HUC12 numbers are changed from time to time. We used a 2009 WBD HUC12 dataset for compatibility with prior work, and this will differ somewhat from currently downloadable HUC datasets. For this reason we include the 2009 WBD HUC12 feature class in our geodatabase, as we used them in defining our transfers, with centroid coordinates added to the attribute table. We also include a feature class of connection lines from origin HUC centroid to destination HUC centroid, each such line being a schematic representation of a step as illustrated in Fig. 4 and described in Table 1. The user can apply *TransferID* and *StepID* as labels, can add directional arrows, or can symbolize by volume or other attributes, as needed. Note that nowhere do we indicate infrastructure location more precisely than HUC12 centroid, so as not to compromise sensitive location information.

The final element of the geodatabase is a toolbox containing two ArcGIS Modelbuilder models to help users build feature subsets consisting of origin and destination HUCs, respectively, and to rebuild step connection lines after adding any new steps or transfers. The user will need to adjust paths in the input and output elements to reflect locations in one’s own computing environment.

### IBT volume data records

**Fig. 5:**
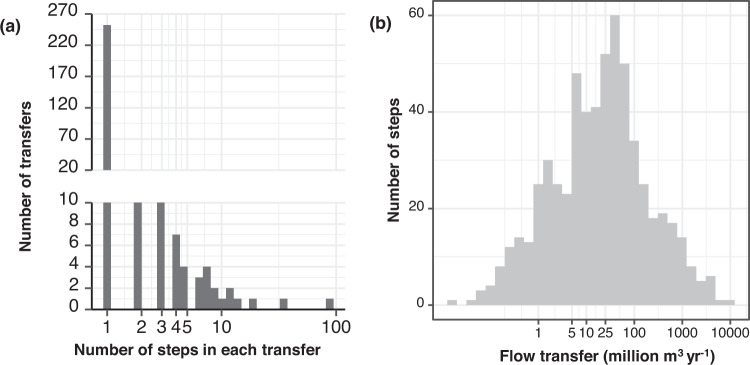
Summary graphs of transfer characteristics. In (**a**), the number of transfers composed of different numbers of steps is shown. In (**b**), the number of steps carrying different volumes of water is shown.

**Fig. 6:**
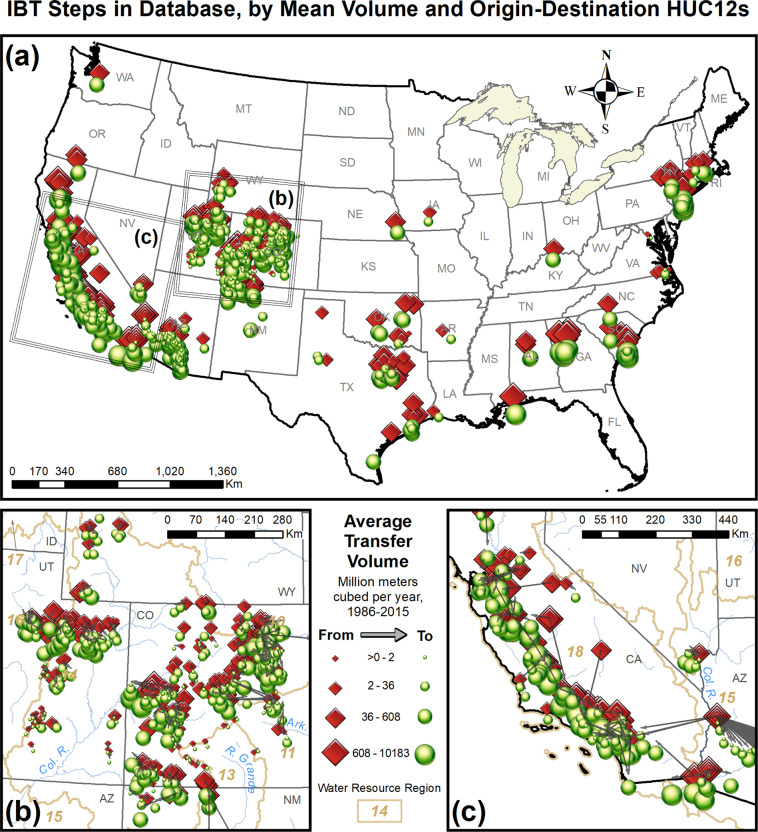
Each inter-basin surface water transfer mapped by average annual volume, 1986–2015. From- and to- points represent origins and destinations of the transfer steps, respectively, but the symbols are offset here for readability. In (**a**), transfers in CONUS are shown. In (**b**) and (**c**), respectively, transfers are shown at more readable scale in the complex area in and adjacent to WRR14 and in the more southerly parts of WRR18.

All transfer volume data reside in the StepVolH12 table in the geodatabase. Each record (row) of the table represents a single step; multi-step transfers thus occupy multiple rows of the table and steps can therefore be modelled individually from that data. Fields in this table include *TransferID*, *StepID*, *FromHUC12*, *ToHUC12*, and fields for annual volumes from 1986 through 2015, in Mm^3^ water yr^−1^. Lastly are several fields for key metadata content about the step, including type of year (i.e., calendar or water year, and how that water year is defined if available) reflected in the data, general and specific data source information to allow users to find the original data we used, comments where needed, an indicator of reservoirs at the beginning and/or end of a step, and transfer type (to surface, into ground, or calculated losses). The StepVolH12 table contains 627 records, 599 of which we acquired data for from at least some years in the 1986–2015 range. For the remaining 28 records, we retained a record toward future data availability. The majority of the records represent single-step transfers, although some of the remaining transfers have dozens of steps (Fig. 5a). The total volume of transferred water represented in the database is 119,909 Mm^3^ water yr^−1^, ranging from 0.01 Mm^3^ water yr^−1^ to 10,182 Mm^3^ water yr^−1^ (Fig. 5b). Figure 6 maps the transfers by average annual volume.

## Technical Validation

We employed two approaches to validate our data: a) cross-checking multiple information and data sources about individual IBTs or IBT systems, and b) pre-testing IBT data in relation to modelled natural water volumes available for transfers.

### Cross-checking multiple sources

We used multiple sources of information to investigate each transfer. These included textual materials describing a PWS, major IBT project, or individual infrastructural component; generalized or schematic maps of PWS water sources or major projects; general reference maps, satellite imagery, and geospatial datasets (notably the NHD); and tabular water volume data. If the tabular data aligned with our understanding from the other source types, the process would stop there. In some cases, however, tabular data suggested we had missed some part of the system, and the process would become iterative. The case of the San Luis Reservoir and associated state-federal joint-use complex is discussed below to illustrate the iterative process of cross-checking source materials sometimes necessary to achieve validated IBT data.

In this example, the San Luis Reservoir is an “off-stream” storage facility that functions as a “regulator”^48^ for both of California’s two massive and intertwined north-to-south water transfer systems, the State Water Project (SWP) (state) and the Central Valley Project (CVP) (federal). The reservoir and associated IBTs make fall-winter-spring water from northern California available for summer irrigation demand in central and southern California as well as year-round demand in California’s southern cities and to some extent buffers the effects of drought years through storage and release cycles. Beginning from this understanding of the system, we sought out SWP and CVP data. From the former source, we used pumping plant data to derive amounts of water flowing into the system from the California Aqueduct (i.e., Banks Pumping Plant minus South Bay Pumping Plant). From the latter source we derived the amount of water in the Delta-Mendota Canal arriving at the system, and the amount actually entering the system (the O’Neill Pumping Plant data category). These two project data sources provided no information about the workings of the regulator system itself, nor any clear data on amounts of water leaving the system. We therefore turned to further examination of textual descriptions and map sources. In doing so we found that the San Luis Reservoir is only one part of the joint-use complex. One reflection of this is the existence of two additional data series covering the joint federal-state operation of the system. Column headings in these joint operations data tables were not self-explanatory, requiring again a return to textual materials and map sources (imagery and elevation data, especially) to understand the system of infrastructure including pumping/generating stations moving the water from one waterbody/elevation to another. We then returned to the data tables and were able to capture the volumes of water moving through the different components of the system with much more completeness and certainty than our original two data sources and superficial understanding allowed.

The iterative process detailed above revealed that new water enters the system at the O’Neill Forebay, from the California Aqueduct without pumping (TransferID 18011.0102) and from the Delta-Mendota Canal after pumping at the O’Neill plant (18012.0101). Water from the O’Neill Forebay reaches the San Luis Reservoir via pumping at the San Luis plant, sometimes called the Gianelli plant (18012.0201). From the reservoir, certain water is delivered to Santa Clara and Hollister, CA (18012.03xx and 18012.0401), removing this water from circulation in the joint-use complex. Remaining water in the San Luis Reservoir is released back to the O’Neill Forebay as needed, again via the San Luis plant but this time generating electricity as it falls (18012.0501). Water released from the Forebay to the Delta-Mendota Canal likewise generates electricity as it falls through the O’Neill plant (18012.0601). Water released from the Forebay to the California Aqueduct (18011.0201) is measured at the Dos Amigos pump to the south. As a final check, we followed our compiled data through the system arithmetically for several random years. Given that our transfer data did not quantify volumes retained for storage, the inflows and outflows of the system balanced each other within reasonable limits and validated our understanding of this complex system.

### Pre-testing IBT data in relation to natural flow directions and volumes

As there is no other comparable IBT database for validation, we indirectly validated the volumes in our IBT dataset using the Water Supply Stress Index (WaSSI) hydrologic model^49^ by comparing the annual transferred volume to the simulated available water supply at each IBT transfer HUC12. The WaSSI model has been validated^49–54^ and used in several regional and national scale water resource assessment studies^24,42,43,53,55,56^. The core of the WaSSI model is an evapotranspiration (ET) empirical model derived from multisite eddy covariance measurements using potential ET^57^, precipitation, and leaf area index (LAI)^58^. Sacramento Soil Moisture Accounting Model (SAC-SMA)^59,60^ was incorporated to further consider the limit of soil moisture on ET demand. Full details of the WaSSI model are well described in refs. ^49,50^.

The WaSSI model is parameterized using readily available national-scale soil, land cover, and climate data. All input datasets were spatially rescaled using an area-weighted averaging scheme to match the scale of analysis (i.e., HUC12 watershed scale). Water yield is calculated for each land cover type in a given HUC12 as the sum of surface runoff from pervious and impervious surfaces, interflow, and baseflow after accounting for losses that include changes in water storage in the soil, evaporation, and transpiration from vegetation. Water yield for each HUC12 is then calculated as the sum of the area-weighted averages of water yield of each land cover type present. Water yield for each HUC12 is then accumulated from upstream to downstream HUC12s along the river network to estimate the total available water supply at the outlet of each respective HUC12. The water supply is the sum of the water yield generated in all HUC12s upstream of a given location on the river network.

To compare the IBT transfers to the simulated natural available water supply of each HUC12, we modified the WaSSI model to account for water transfers through IBTs, incorporating the water transferred from the source to the destination HUC12 for all IBTs in the flow accumulation calculations. Thus the annual total available water supply for each HUC12 equals the simulated natural available water supply plus the simulated net difference made by including IBT data in the WaSSI model. Where a negative annual total available water supply was returned, but there was a reservoir present, we assumed that the net difference would be supplied from the reservoir. We found 17 HUC12s which yielded a negative annual total available water supply in various years, but which did *not* have a reservoir to supply the difference. Where this situation occurred in our pretesting, we checked data source and data entry, and looked for possible IBTs not previously discovered. We found one such IBT; for the remainder we found that one or more IBT-in had a value of NoData for the year(s) of concern. Since NoData does not indicate zero flow, only an absence of data, we assumed there actually was sufficient flow in these cases.

## Usage Notes

The table StepVolH12 contains the key data for modelling; users may wish to export the table to another format for inclusion in complex calculations.

Where an agency or source did not supply any data for a given year and given step, we used a value of −999 as the NoData value. If the agency reported a volume of zero, we used a value of 0 in the table.

For users unfamiliar with ArcGIS Modelbuilder, we suggest you use ArcCatalog to view and run the supplied models. Open model with the Edit option. Modify the source and output paths and/or names before running the model.

## Supplementary information

Supplemtary Table 1

## Data Availability

The geodatabase was created in ArcGIS Desktop 10.5 and 10.7 software by Environmental Systems Research Institute (ESRI). Transfer volume unit conversions were performed in Microsoft Excel. Figures were created using ArcGIS Desktop, X-Ray for Geodatabases 2018.3.12 by Vertex3, Microsoft Visio Professional 2016, and R. The data pre-testing used the WaSSI model which is available as an online tool at https://web.wassiweb.fs.usda.gov/s. We customized the model code in R, and this code is available at https://github.com/ln1267/IBT_Flow_check.

## References

[1] Baires SE (2015). The role of water in the emergence of the pre‐Columbian Native American city Cahokia. WIREs Water.

[2] Barber M, Jackson S (2015). Remembering ‘the blackfellows’ dam’: Australian Aboriginal water management and settler colonial riparian law in the upper Roper River, Northern Territory. Settl. Colon. Stud..

[3] Damp JE, Hall SA, Smith SJ (2002). Early Irrigation on the Colorado Plateau near Zuni Pueblo, New Mexico. Am. Antiq..

[4] Doolittle WE (1995). Indigenous development of Mesoamerican Irrigation. Geogr. Rev..

[5] Du, P. & Koenig, A. in *Evolution of Water Supply through the Millennia* (eds. Angelakis, A. N., Mays, L. W., Koutsoyiannis, D., & Mamassis, N.) History of water supply in pre-modern China 169–226 (IWA Publishing, 2012).

[6] Erickson CL (1992). Prehistoric landscape management in the Andean highlands: raised field agriculture and its environmental impact. Popul. Environ..

[7] Huckleberry G (2012). A non-riverine prehistoric canal system on the Tortolita Mountain piedmont, Southern Arizona. KIVA.

[8] Khan S, Yilmaz N, Valipour M, Angelakis AN (2021). Hydro-technologies of Mehrgarh, Baluchistan and Indus Valley Civilizations, Punjab, Pakistan (ca. 7000–1500 BC). Water.

[9] Lizarzaburu J (2020). The Surco canal, an ancient irrigation canal in Lima, Peru, and a citizens’ campaign for its protection. Water Hist..

[10] Mahmoudian, S. A. & Mahmoudian, S. N. in *Evolution of Water Supply through the Millennia* (eds. Angelakis, A. N., Mays, L. W., Koutsoyiannis, D., & Mamassis, N.) Water and water supply technologies in ancient Iran 92–127 (IWA Publishing, 2012).

[11] Masse WB (1981). Prehistoric irrigation systems in the Salt River valley, Arizona. Science.

[12] Matsui K, Berry K, Cohn TC, Jackson S (2016). Indigenous water histories I: recovering oral histories, interpreting Indigenous perspectives, and revealing hybrid waterscapes. Water Hist..

[13] Mays LW, Koutsoyiannis D, Angelakis AN (2007). A brief history of urban water supply in antiquity. Water Supply.

[14] Nichols DL (1982). A Middle Formative irrigation system near Santa Clara Coatitlan in the Basin of Mexico. Am. Antiq..

[15] Prümers H, Betancourt CJ, Iriarte J, Robinson M, Schaich M (2022). Lidar reveals pre-Hispanic low-density urbanism in the Bolivian Amazon. Nature.

[16] Purdue L, Salomon F, Berger J-F, Goiran J-P (2015). Canal through time: towards a multidisciplinary and holistic study of water systems?. Water Hist..

[17] Rose D, Bell D, Crook DA (2016). Restoring habitat and cultural practice in Australia’s oldest and largest traditional aquaculture system. Rev. Fish Biol. Fisheries.

[18] Salomon F, Purdue L, Goiran J-P, Berger J-F (2014). Introduction to the special issue: Roman canals studies—main research aims. Water Hist..

[19] Turner BL, Harrison PD (1981). Prehistoric raised-field agriculture in the Maya lowlands. Science.

[20] Ertsen MW (2010). Structuring properties of irrigation systems: understanding relations between humans and hydraulics through modeling. Water Hist..

[21] Hayashida FM (2006). The Pampa de Chaparrí: water, land, and politics on the north coast of Peru. Lat. Am. Antiq..

[22] Zhuang W (2016). Eco-environmental impact of inter-basin water transfer projects: a review. Environ Sci. Pollut. Res..

[23] Brown TC, Mahat V, Ramirez JA (2019). Adaptation to future water shortages in the United States caused by population growth and climate change. Earth’s Future.

[24] Duan K (2019). Understanding the role of regional water connectivity in mitigating climate change impacts on surface water supply stress in the United States. J. Hydrol..

[25] Duan, K. *et al*. Climate change challenges efficiency of inter-basin water transfers in alleviating water stress. *Environ. Res. Lett*. **17** (2022).

[26] Emanuel RE, Buckley JJ, Caldwell PV, McNulty SG, Sun G (2015). Influence of basin characteristics on the effectiveness and downstream reach of interbasin water transfers: displacing a problem. Environ. Res. Lett..

[27] Mooty, W. S. & Jeffcoat, H. H. *Inventory of interbasin transfers of water in the eastern United States*. Report No. 86–148 (United States Geological Survey, 1986).

[28] Petsch, H. E. Jr. *Inventory of interbasin transfers of water in the western conterminous United States*. Report No. 86–166 (United States Geological Survey, 1985).

[29] Dickson KE, Dzombak DA (2017). Inventory of interbasin transfers in the United States. J. Am. Water Resour. Assoc..

[30] Zhang L, Li S, Loáiciga HA, Zhuang Y, Du Y (2015). Opportunities and challenges of interbasin water transfers: a literature review with bibliometric analysis. Scientometrics.

[31] Davies BR, Thoms M, Meador M (1992). An assessment of the ecological impacts of inter-basin water transfers, and their threats to river basin integrity and conservation. Aquat. Conserv..

[32] Snaddon CD, Wishart MJ, Davies BR (1998). Some implications of inter-basin transfers for river ecosystem functioning and water resources management in southern Africa. Aquat. Ecosyst. Health Manag..

[33] Matete M, Hassan R (2006). Integrated ecological economics accounting approach to evaluation of inter-basin water transfers: an application to the Lesotho Highlands Water Project. Ecol. Econ..

[34] Fornarelli R, Galelli S, Castelletti A, Antenucci JP, Marti CL (2013). An empirical modeling approach to predict and understand phytoplankton dynamics in a reservoir affected by interbasin water transfers: empirical modeling of phytoplankton. Water Resour. Res..

[35] Gallardo B, Aldridge DC (2018). Inter-basin water transfers and the expansion of aquatic invasive species. Water Res..

[36] Islar, M. & Boda, C. Political ecology of inter-basin water transfers in Turkish water governance. *Ecol. Soc*. **19** (2014).

[37] Garrick D (2019). Rural water for thirsty cities: a systematic review of water reallocation from rural to urban regions. Environ. Res. Lett..

[38] Berry KA, Jackson S, Cohn TC, Matsui K (2017). Indigenous water histories II: water histories and the cultural politics of water for contemporary Indigenous groups. Water Hist..

[39] Dickson KE, Dzombak DA (2019). Drivers of interbasin transfers in the United States: insights from sampling. J. Am. Water Resour. Assoc..

[40] McDonald RI (2014). Water on an urban planet: urbanization and the reach of urban water infrastructure. Glob. Environ. Change.

[41] Purvis L, Dinar A (2020). Are intra- and inter-basin water transfers a sustainable policy intervention for addressing water scarcity?. Water Secur..

[42] Liu N (2021). Forested lands dominate drinking water supply in the conterminous United States. Environ. Res. Lett..

[43] Liu, N. *et al*. Inter‐basin transfers extend the benefits of water from forests to population centers across the conterminous U.S. *Water Resour. Res*. **58** (2022).

[44] Hornberger GM, Hess DJ, Gilligan J (2015). Water conservation and hydrological transitions in cities in the United States: municipal water conservation. Water Resour. Res..

[45] Kummu M (2016). The world’s road to water scarcity: shortage and stress in the 20th century and pathways towards sustainability. Sci. Rep..

[46] Padowski, J. C. & Jawitz, J. W. Water availability and vulnerability of 225 large cities in the United States: urban water availability and vulnerability. *Water Resour. Res*. **48** (2012).

[47] Dobbs GR, Liu N, Caldwell PV (2023). figshare.

[48] California Department of Water Resources. *Initial Study of the Long-Term Operation of the State Water Project*. (2019).

[49] Sun G (2011). Upscaling key ecosystem functions across the conterminous United States by a water-centric ecosystem model. J. Geophys. Res..

[50] Caldwell PV, Sun G, McNulty SG, Cohen EC (2012). & Moore Myers, J. A. Impacts of impervious cover, water withdrawals, and climate change on river flows in the conterminous US. Hydrol. Earth Syst. Sci..

[51] Caldwell PV (2015). A comparison of hydrologic models for ecological flows and water availability: model comparison for ecological flow and water availability. Ecohydrol..

[52] Caldwell, P. V. *et al*. *Hydrologic modeling for flow-ecology science in the southeastern United States and Puerto Rico*. e-Gen. Tech. Rep. SRS-246 (U.S. Department of Agriculture Forest Service, Southern Research Station, 2020).

[53] Li, C. *et al*. Impacts of urbanization on watershed water balances across the conterminous United States. *Water Resour. Res*. **56** (2020).

[54] Schwalm CR (2015). How well do terrestrial biosphere models simulate coarse-scale runoff in the contiguous United States?. Ecol. Modell..

[55] Duan K, Sun G, Caldwell PV, McNulty SG, Zhang Y (2018). Implications of upstream flow availability for watershed surface water supply across the conterminous United States. J. Am. Water Resour. Assoc..

[56] Vose JM (2016). Ecohydrological implications of drought for forests in the United States. For. Ecol. Manag..

[57] Hamon, W. R. Computation of direct runoff amounts from storm rainfall. *Int. Assoc. Sci. Hydrol. Publ*. **63**, 52–62.

[58] Sun G (2011). A general predictive model for estimating monthly ecosystem evapotranspiration. Ecohydrol..

[59] Burnash, R. J. C. *A Generalized Streamflow Simulation System: Conceptual Modeling for Digital Computers*. (U. S. Department of Commerce, National Weather Service, and State of California, Department of Water Resources, 1973).

[60] Burnash, R. J. C. in *Computer Models of Watershed Hydrology* (ed. Singh, V. P.) The NWS river forecast system - catchment modelling 311–366 (Water Resource Publications, 1995).

